# Spatial Positioning and Chemical Coupling in Coacervate‐in‐Proteinosome Protocells

**DOI:** 10.1002/anie.201903756

**Published:** 2019-05-22

**Authors:** Richard Booth, Yan Qiao, Mei Li, Stephen Mann

**Affiliations:** ^1^ Centre for Protolife Research and Centre for Organized Matter Chemistry School of Chemistry University of Bristol Bristol BS8 1TS UK

**Keywords:** coacervates, enzymes, membranes, proteinosomes, self-assembly

## Abstract

The integration of molecularly crowded microenvironments into membrane‐enclosed protocell models represents a step towards more realistic representations of cellular structure and organization. Herein, the membrane diffusion‐mediated nucleation of either negatively or positively charged coacervate microdroplets within the aqueous lumen of individual proteinosomes is used to prepare nested hybrid protocells with spatially organized and chemically coupled enzyme activities. The location and reconfiguration of the entrapped droplets are regulated by tuning the electrostatic interactions between the encapsulated coacervate and surrounding negatively charged proteinosome membrane. As a consequence, alternative modes of a cascade reaction involving membrane‐ and coacervate‐segregated enzymes can be implemented within the coacervate‐in‐proteinosome protocells.

Micro‐compartmentalized systems enriched with biomimetic functions offer a bottom‐up approach to the chemical construction of synthetic protocells.^1^, ^2^ To date, the design of artificial cells has focused primarily on two key architectural modes:^3^ i) semi‐permeable membrane‐enclosed spherical assemblies such as vesicles prepared from lipids^4^, ^5^, ^6^ or polymers,^7^, ^8^ water droplet‐in‐oil emulsions,^9^, ^10^ inorganic nanoparticle‐stabilized colloidosomes,^11^, ^12^ and protein‐polymer microcapsules (proteinosomes);^13^, ^14^ and ii) membrane‐free droplets such as coacervates or soft colloidal microgels that comprise molecularly crowded interiors capable of sequestering diverse molecular and macromolecular components.^15^, ^16^, ^17^, ^18^, ^19^ Recently, hybrid protocells based on combinations of these two modules have been explored as a step towards more realistic models of cell‐like structure and organization. For example, the spontaneous assembly of fatty acids,^20^ block copolymers,^21^ or silica nanoparticles^22^ on the surface of preformed coacervate microdroplets has been used to prepare membrane‐coated molecularly crowded hybrid protocells. Coacervate‐in‐liposome architectures have been prepared by in situ phase transformation using temperature‐responsive polymers^23^ or changes in temperature and osmotic pressure.^24^, ^25^ Interactions between populations of preformed coacervate microdroplets and proteinosomes have been explored as pathways to artificial predation^26^ and phagocytosis.^27^ In the latter case, spontaneous transfer of single proteinosomes across the interface of large coacervate droplets produced hybrid protocells lacking an outer membrane.

Herein, we describe the fabrication of nested coacervate‐in‐proteinosome protocells by chemically induced coacervation within the aqueous lumen of individual proteinosomes. For this study, we prepare proteinosomes with encapsulated polyelectrolytes and add counter‐charged membrane‐permeable small molecules to the external aqueous environment. Diffusion of the small molecules across the protein‐polymer membrane gives rise to in situ complexation and electrostatically induced formation of a coacervate phase. The spatial location and reconfiguration of the entrapped microdroplets are regulated by tuning the electrostatic interactions between the encapsulated coacervate and surrounding proteinosome membrane to produce nested hybrid protocells with spatially organized and chemically coupled enzyme activities.

We developed the above strategy using proteinosomes with a negatively charged membrane (−9 mV; see Figure S1 in the Supporting Information) based on a crosslinked monolayer of bovine serum albumin/poly(N‐isopropylacrylamide (BSA‐NH_2_/PNIPAAm) nanoconjugates.^13^, ^14^ A coacervate phase with negative surface potential (−16 mV, see Figure S1) was nucleated within proteinosomes containing fluorescein isothiocyanate labelled carboxymethyldextran (FITC‐CMD, M_W_≈70 k) by addition of the highly positively charged antimicrobial agent chlorhexidine (CHXD, M_W_≈701) to the external solution phase at pH 8 and FITC‐CMD:CHXD monomer molar ratio of 2:1 (Figure 1 a). Formation of the entrapped coacervate phase occurred within seconds of mixing, indicating unhindered diffusion of CHXD through the protein‐polymer membrane and strong binding of the antimicrobial to the encapsulated polysaccharide molecules. Microscopy images of samples prior to addition of CHXD showed optically transparent proteinosomes with uniform green fluorescence, consistent with a homogeneous distribution of FITC‐CMD throughout the aqueous lumen (Figure 1 b,c). In contrast, after addition of CHXD the nested protocells exhibited higher optical contrast and discrete localized regions of green fluorescence, consistent with the nucleation and entrapment of a condensed FITC‐CMD/CHXD coacervate phase (Figure 1 d,e; see Figure S2). Confocal fluorescence microscopy images of single proteinosomes confirmed the presence of a high‐density population of non‐coalesced coacervate microdroplets throughout the proteinosome interior (Figure 1 f; see Figure S3). The entrapped droplets were 0.5–2.5 μm in diameter, which was similar to the range of sizes observed in bulk solution (0.5–4.25 μm; see Figure S4). Fluorescence microscopy video imaging indicated that the entrapped coacervate droplets were in constant flux at room temperature (see Movies S1 and S2), suggesting only a minimal level of interaction with the negatively charged proteinosome membrane.

**Figure 1:**
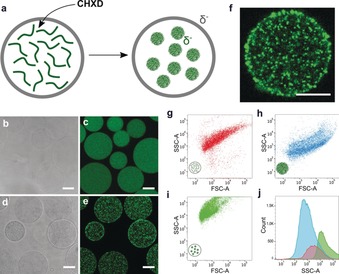
a) Scheme illustrating the formation of coacervate‐in‐proteinosome nested protocells. Diffusion of CHXD into pre‐assembled proteinosomes (left graphic) containing FITC‐CMD (green lines) gives rise to in situ complexation and electrostatically induced formation of FITC‐CMD/CHXD coacervate microdroplets (green filled circles) within the host protocell (right graphic). Both the proteinosome membrane and coacervate droplets are negatively charged (δ−). b–e) Optical (b,d) and confocal fluorescence (c,e) microscopy images of proteinosomes containing FITC‐CMD before (b,c) and after (d,e) addition of CHXD showing changes in optical texture and fluorescence distribution associated with in situ coacervate droplet assembly; scale bars=20 μm. f) Confocal fluorescence microscopy image of a single proteinosome showing dispersed population of discrete green‐fluorescent FITC‐CMD/CHXD coacervate microdroplets within the protocell microcompartment; scale bar=20 μm. g–i) FACS‐derived 2D dot plots of side‐scattered light area (SSC‐A) versus forward‐scattered light area (FSC‐A) for proteinosomes containing only CMD‐FITC (g), bulk FITC‐CMD/CHXD coacervate micro‐droplets (h), and nested coacervate‐in‐proteinosome protocells (i). The number of particles analyzed in each sample was between 10–20×10^3^. j) Corresponding histograms showing number of counts against SSC‐A values determined for samples (g), (h), and (i) (red, blue, and green, respectively), showing significant increases in the SSC‐A values associated with the hybrid protocells.

Statistical measurements of the in situ assembly process were undertaken using fluorescence activated cell‐sorting (FACS). Individual populations of FITC‐CMD‐containing proteinosomes and bulk samples of FITC‐CMD/CHXD coacervate microdroplets gave two‐dimensional (2D) scattering dot plots that were clearly distinguishable (Figure 1 g,h). Addition of CHXD to a dispersion of the proteinosomes afforded a new population with significantly higher side‐scattered light area (SSC‐A) values than either parent population (Figure 1 i). As coacervate formation in the external medium was associated with significantly lower SSC‐A values (Figure 1 j), the new scattering profile was attributed to an increase in optical granularity arising from formation of the coacervate‐in‐proteinosome nested microarchitecture.

A similar strategy of diffusion‐mediated chemically induced complexation was used to prepare nested protocells comprising a negatively charged proteinosome outer membrane and entrapped coacervate phase with a slightly positive surface potential (+3 mV; see Figure S5). We reasoned that attractive electrostatic interactions between the two types of co‐located protocells could offer a mechanism for controlling the spatial positioning and chemical coupling of the entrapped coacervate phase and associated enzyme payloads (Figure 2 a). To achieve this control, membrane‐permeable adenosine 5′‐triphosphate (ATP) was added to an aqueous dispersion of proteinosomes containing the cationic polymer poly(diallyldimethyl‐ammonium chloride (PDDA, M_W_=100–200 kDa; ATP:PDDA=1:1) to produce a coacervate phase within a few minutes. DyLight 405 labelled glucose oxidase (GOx) was co‐encapsulated into the proteinosomes to provide a fluorescence probe for determining the spatial location of the coacervate phase given that the measured GOx equilibrium partition constant in the coacervate phase (*K*_coac_) was large (*K*_coac_=57). Optical and fluorescence microscopy images indicated that unlike the negatively charged FITC‐CMD/CHXD droplets (Figure 1 f), the slightly positive GOx‐containing ATP/PDDA coacervate phase was spatially distributed along the inner surface of the negatively charged proteinosome membrane in the form of a thin submembrane layer typically a few micrometres in thickness (Figure 2 b,c; see Figure S6). Interestingly, the thin coacervate shell could be reconfigured into a homogeneous dispersion of entrapped GOx‐containing coacervate microdroplets by judiciously increasing the ionic strength to disrupt the weak attractive forces operating at the membrane surface without disassembling the coacervate complex (Figure 2 d,e; see Figure S7).

**Figure 2:**
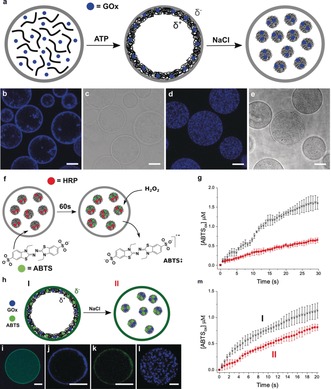
a) Scheme illustrating the spatial positioning and relocation of proteinosome‐entrapped coacervates. Diffusion of ATP into pre‐assembled proteinosomes (left graphic) containing PDDA (black lines) and GOx (filled blue circles) gives rise to the in situ assembly of a positively charged (δ+) GOx‐loaded ATP/PDDA coacervate phase against the negatively charged (δ−) inner surface of the proteinosome membrane (center graphic). Subsequent addition of NaCl results in transformation and relocation of the enzyme‐containing coacervate shell into discrete GOx‐loaded coacervate micro‐droplets dispersed within the proteinosome lumen (right graphic); b,c) Confocal (b) and optical (c) microscopy images of PDDA‐containing proteinosomes after addition of ATP showing formation of a thin sub‐membrane coacervate layer. Blue fluorescence arises from DyLight 405‐GOx sequestered into the coacervate phase; scale bars=20 μm. d,e) As for b,c but after addition of NaCl showing relocation of the coacervate phase into micro‐droplets dispersed throughout the lumen; scale bars=20 μm. f) Scheme illustrating sequestration of encapsulated HRP (red circles) into proteinosome‐entrapped ATP/PDDA coacervate droplets followed by addition of ABTS (green circles) to the external solution and diffusion of the substrate across the proteinosome membrane. After sequestration of ABTS into the coacervate phase (60 s), H_2_O_2_ is added to the external solution to initiate HRP‐mediated oxidation of ABTS in the entrapped coacervate droplets. A similar reaction was undertaken with nested protocells containing a coacervate sub‐membrane shell. g) Time profile showing changes in concentration of oxidized ABTS ([ABTSox]) associated with the reaction Scheme shown in f in the presence (black) and absence (red) of entrapped ATP/PDDA coacervate micro‐droplets. Error bars in g represent the standard deviation of [ABTSox] produced from three separate experiments. h) Scheme illustrating spatial coupling of coacervate/proteinosome GOx/HRP enzyme cascades in nested hybrid protocells. Two alternative arrangements (I and II) are shown. In each case, a semipermeable crosslinked HRP‐containing proteinosome membrane (dark green circle) is employed to house an ATP/PDDA coacervate phase loaded with GOx (filled blue circles) and ABTS (filled green circles). The enzyme and substrate are positioned either in a thin coacervate shell located directly on the HRP‐active membrane (I) or within an entrapped dispersion of coacervate microdroplets (II). Addition of glucose to the external solution initiates the cascade reaction in both cases. i) Confocal fluorescence microscopy image of a single native proteinosome comprising a FITC‐HRP‐active membrane and encapsulated PDDA and DyLight 405‐GOx. A green fluorescent HRP membrane and homogeneous blue fluorescent GOx interior is observed in the absence of ATP. j,k) As for i but after addition of ATP and recorded as blue‐ (j) or green‐ (k) filtered images, showing the presence of the GOx‐loaded coacervate phase directly against the inner surface of the HRP‐containing proteinosome membrane. l) As for j, but after addition of NaCl showing re‐location of the GOx/coacervate phase into droplets away from the HRP‐containing membrane. All scale bars=20 μm. m) Time profile showing increase in [ABTSox] for GOx/HRP cascade reactions in nested coacervate‐in‐proteinosome protocells organized in arrangements I or II. Error bars in (m) represent the standard deviation of [ABTSox] produced from three separate experiments.

We exploited the positioning of coacervate‐sequestered enzymes under the protein‐polymer membrane or within the aqueous lumen to modulate the catalytic activity of the nested hybrid protocells (Figure 2 f). Specifically, we encapsulated horseradish peroxidase (HRP) in the PDDA‐containing proteinosomes, added ATP in the external solution to generate the entrapped slightly positively charged coacervate phase, and then added the HRP substrate, 2′‐azino‐bis(3‐ethylbenzothiazoline‐6‐sulphonic acid) (ABTS), to the external environment. Diffusion of ABTS across the proteinosome membrane resulted in co‐sequestration of the substrate (*K*_coac_=60) and HRP (*K*_coac_=68) in the ATP/PDDA coacervate phase. Subsequent addition of hydrogen peroxide (H_2_O_2_) to the external solution followed by diffusion across the proteinosome membrane initiated the one‐electron oxidation of ABTS specifically within the entrapped coacervate microdroplets. The corresponding initial rate of increase in absorbance at 410 nm, associated with formation of the oxidized ABTS radical cation, was several times faster in both the coacervate shell and droplet configurations compared with coacervate‐free proteinosomes containing PDDA, HRP, and ABTS (Figure 2 g; see Figures S8 and S9). The initial rates of reaction at 25 °C were 47±8, 76±12, and 28±2 nm s^−1^ respectively. The results indicated that the hybrid microsystems were considerably more reactive compared with the HRP‐containing coacervate‐free proteinosomes, consistent with similar enhancements observed in previously studied bulk coacervate systems.^15^, ^18^, ^28^

Given the above observations we exploited the different spatial configurations of the nested protocell architecture to implement alternative modes of an enzyme cascade reaction. Specifically, a coupled GOx/HRP enzyme cascade was established between the proteinosome membrane and entrapped ATP/PDDA coacervate phase. For this step, we prepared proteinosomes in which HRP‐NH_2_/PNIPAAm nanoconjugates were co‐assembled and crosslinked into the BSA‐NH_2_/PNIPAAm protein‐polymer membrane, and GOx and ABTS sequestered into the two alternative spatial arrangements (thin submembrane shell or dispersed droplets) of the entrapped coacervate phase (Figure 2 h). In both cases, addition of glucose resulted in a two‐step GOx/HRP cascade reaction involving the coacervate‐induced production of H_2_O_2_, diffusion of the peroxide signal from the coacervate phase to the HRP‐active proteinosome membrane, and oxidative processing of ABTS. Confocal fluorescence microscopy images confirmed the presence of HRP and GOx throughout the proteinosome membrane and aqueous PDDA‐containing lumen, respectively, prior to addition of ATP (Figure 2 i). Addition of ATP followed by ABTS resulted in translocation of GOx into a thin coacervate shell positioned against the inner surface of the HRP‐containing membrane (Figure 2 j,k). As a consequence, the two enzymes of the cascade reaction were immobilized in close spatial proximity. Alternatively, the spatial separation between the catalytic partners could be increased by adding NaCl, which disassembled the coacervate shell to produce an entrapped dispersion of GOx‐containing ATP/PDDA microdroplets (Figure 2 l). Corresponding measurements of the reaction rates recorded over an initial 4 second period after addition of glucose gave values of about 115 and 48 nm s^−1^ at 25 °C when GOx and ABTS were located within the thin coacervate shell or dispersed droplets, respectively (Figure 2 m). We attributed the threefold kinetic enhancement to the closer spatial and diffusive coupling of the cascade reaction at the HRP‐containing membrane such that H_2_O_2_ and ABTS were more effectively channeled between the two enzymes in the early stages of the reaction. In contrast, the reaction rates became comparable at later time stages (Figure 2 m) as diffusive mixing became predominant, in agreement with previous studies on enzyme channelling.^29^, ^30^

Herein, we describe a facile in situ route to the spontaneous formation of coacervate‐in‐proteinosome protocells as a step towards a new hybrid protocell model that integrates structural and functional aspects of membrane enclosure and molecular crowding. By encapsulating either positively or negatively charged polyelectrolytes within the proteinosomes, we exploit the diffusion of counter‐charged small molecules through the semipermeable protein‐polymer membrane to initiate chemically induced coacervation and positioning of sequestered enzymes and enzyme substrates within the aqueous lumen. This strategy enables micro‐compartmentalized coacervation to occur without the requirement for temperature‐ or osmotic‐pressure‐induced phase transformation,^23^, ^24^, ^25^ offering increased operational flexibility provided that the molecularly crowded phase can be prepared using a membrane‐permeable constituent. If required, coacervate‐in‐proteinosomes can also be prepared using mixtures of membrane‐impermeable polyelectrolytes by co‐encapsulation and in situ salt‐induced phase transformation (see the Supporting Information).

In principle, the formation of multi‐tiered protocells capable of concentrating and organizing different functional components into distinct spatial arrangements in a reversible manner provides an opportunity to develop artificial cell models comprising specialized subcompartments (proto‐organelles). In this regard, proteinosome‐enclosed coacervation offers several advantages based on the ease of fabrication, high sequestration potential, and ability to segregate enzymes in both the membrane and interior, which together provide a flexible platform for developing rudimentary artificial metabolic reaction networks for energy capture, chemical storage, and inter‐protocell signaling. Moreover, as DNA components can be sequestered into coacervate droplets^26^ and used as logic gates in proteinosome communication pathways,^31^ integration of DNA nanotechnology into coacervate‐in‐proteinosome microarchitectures might facilitate the development of lifelike objects with simple forms of embodied chemical computation.

## Conflict of interest

The authors declare no conflict of interest.

## Supporting information

As a service to our authors and readers, this journal provides supporting information supplied by the authors. Such materials are peer reviewed and may be re‐organized for online delivery, but are not copy‐edited or typeset. Technical support issues arising from supporting information (other than missing files) should be addressed to the authors.

Supplementary

Supplementary

Supplementary
