# Supplementary material for: Spatial Positioning and Chemical Coupling in Coacervate‐in‐Proteinosome Protocells
Source: Angew Chem Int Ed Engl. 2019 May 22;58(27):9120–4. doi: 10.1002/anie.201903756 (PMC6618027; doi:10.1002/anie.201903756)
Supplement: Supplementary file 1 — Supplementary [file ANIE-58-9120-s001.pdf]

Angewandte Chemie
Eine Zeitschrift der Gesellschaft Deutscher Chemiker

Supporting Information

Spatial Positioning and Chemical Coupling in Coacervate-in-Proteinosome Protocells

Richard Booth, Yan Qiao, Mei Li, and Stephen Mann**

anie_201903756_sm_miscellaneous_information.pdf
anie_201903756_sm_MovieS1.avi
anie_201903756_sm_MovieS2.avi

Supporting Information

Supplementary videos

Supplementary Movie S1. Confocal fluorescence microscopy video showing the movement of CMD-FITC/CHXD coacervate droplets trapped inside a single BSA-NH_2/PNIPAAm proteinosome. Movie was taken at 10 frames per second and for a total duration of 1.4 s. Scale bar = 20 μm.

Supplementary Movie S2. Confocal fluorescence images of slices across the z-axis of a single BSA-NH_2/PNIPAAm proteinosome showing randomly moving CMD-FITC/CHXD coacervate droplets throughout the aqueous lumen. Slices are approximately 0.22 μm apart; scale bar = 20 μm.

Supplementary Methods

Preparation of BSA-NH_2/PNIPAAm nanoconjugates

Cationized bovine serum albumin (BSA-NH_2) was synthesized according to a previously reported method [Huang, X.; Li, M.; Green, D. C.; Williams, D. S.; Patil, A. J.; Mann, S. Nat. Commun. 2013, 4, 2239 DOI: 10.1038/ncomms3239]. Carbodiimide-activated conjugation was used to attach 1,6-hexanediamine (HMDA) to aspartic and glutamic acid residues on the external surface of BSA. Typically, a solution of HMDA (1000 mg, 8.60 mmol) was adjusted to pH 6.2 using 5 M HCl and added dropwise to a stirred solution of the BSA (100 mg, 1.49 μmol). The pH was readjusted to 6.5 if necessary. The coupling reaction was initiated by adding 100 mg 1-ethyl-3-(3-dimethylaminopropyl)carbodiimide (EDC) immediately and again (100 mg) after 5 h. The pH value was maintained at 6.5 by adding 5 M HCl, and the solution was stirred overnight, and then the solution was centrifuged at 5000 rpm for 5 mins, the supernatant taken and any precipitate discarded. The BSA-NH_2-containing supernatant was dialyzed (Medicell, dialysis tubing, MWCO 12−14 kDa) extensively against Milli-Q water, and freeze-dried.

End-capped mercaptothiazoline-activated PNIPAAm (Mn = 8800 g mol^{-1}, 20 mg in 10 mL of water) was synthesized according to the previously reported method [Huang, X.; Li, M.; Green, D. C.; Williams, D. S.; Patil, A. J.; Mann, S. Nat. Commun. 2013, 4, 2239 DOI: 10.1038/ncomms3239]. Synthesised PNIPAAm was then added to a stirred solution of BSA-NH_2 (20 mg in 10 mL of sodium carbonate buffer at pH 8.5). The mixed solution was stirred for 12 h and then purified by using a centrifugal filter (Millipore, Amicon Ultra, MWCO 50 kDa) to remove any unreacted PNIPAAm and salts. After freeze-drying, the BSA-NH_2/PNIPAAm conjugate was obtained. Based on MALDI-Tof and UV-vis spectroscopy measurements, there were on average 3.4 PNIPAAm chains covalently coupled to each BSA molecule. The same procedure was carried out on HRP, an average of 2 PNIPAAm chains were found to be coupled to each HRP molecule.

Preparation of BSA-NH_2/PNIPAAm proteinosomes

BSA-NH_2/PNIPAAm proteinosomes were prepared according to a previously described protocol [Huang, X.; Li, M.; Green, D. C.; Williams, D. S.; Patil, A. J.; Mann, S. Nat. Commun. 2013, 4, 2239 DOI: 10.1038/ncomms3239]. Briefly, 1.5 mg of PEG-bis(N-succinimidyl succinate) cross-linker was added to 60 μL of a 4.0 mg mL^{-1} BSA-NH_2/PNIPAAm solution in 50 mM carbonate buffer (pH 8.5), immediately followed by the addition of 1 mL of 2-ethyl-1-hexanol. The mixture was gently hand-shaken for 10s to produce a protein-polymer stabilized water-in-oil emulsion (aqueous/oil volume fraction, Φ_w, of 0.06). The cross-linker was allowed to react with the primary amine groups in BSA-NH_2 at the water-oil interface for 48 h. After 48 h sedimentation, the upper clear oil layer was discarded, and 1 mL of a 65% ethanol/water mixture added to dissolve the sediment. The solution

was then dialyzed against 65%, 40% and 20% ethanol/water for 2 h, then against Milli-Q water for 1 day to complete the transfer of the cross-linked proteinosomes into water.

Preparation of polyelectrolyte-containing proteinosomes
Proteinosomes were prepared by the spontaneous assembly of synthesized BSA-NH_2/PNIPAAm nanoconjugates at the interface of water-in-2-ethyl-1-hexanol emulsion droplets (see above). Typically, negatively charged CM-dextran (labelled with FITC, M_W = 70 kDa) or positively charged PDDA (M_W = 100-200 kDa) was encapsulated within the proteinosomes by mixing an aqueous solution of each polyelectrolyte (5 µL, 120 mM (monomer concentration)) with an aqueous solution of BSA-NH_2/PNIPAAm nanoconjugates (30 µL, 8 mg.mL^{-1}, 50 mM carbonate buffer, pH 8.5) containing a PEG-bis(N-succinimidyl succinate) cross-linker (1.2 mg). The mixture was then added to 1 mL of 2-ethyl-1-hexanol oil (1 mL) and the samples shaken by hand for 10 s and left to stand for 48 h. After 48 h sedimentation, the upper clear oil layer was discarded, and 1 mL of a 65 vol% ethanol/water mixture added to dissolve the sediment. The solution was then dialyzed against 65, 40 and 20 vol% ethanol/water for 2 h, then against Milli-Q water for 1 day to complete the transfer of the cross-linked proteinosomes into water. Proteinosomes containing 2 : 3 volume mixtures of aqueous PDDA (6.67 µL, 10 mM, M_W = 100-200 kDa) and PAA (10 µL, 10 mM, M_W = 100-200 kDa) in aqueous NaCl (10 µL, 3 M (final concentration, 0.5 M) were prepared as above but in the absence of CM-dextran and PDDA.

Trace amounts of polyelectrolytes in the external water phase accompanying adventitious fragmentation of the proteinosomes during work-up were removed by graduated centrifugation. For this, 1 mL dispersions were centrifuged at 800 rpm for 1 min, followed by further centrifugation at 2000 rpm for 1 min, removal of the supernatant (400 µL) and replacement with 400 µL of Milli-Q water. This process was repeated three times to ensure removal of all extraneous components.

Polyelectrolyte-containing proteinosomes comprising a mixture of membrane building blocks were prepared as above but using a mixture of BSA-NH_2/PNIPAAm and HRP-NH_2/PNIPAAm-FITC nano-conjugates (20 and 80 wt%, respectively).

Preparation of coacervate-in-proteinosome nested protocells
A CMD-FITC/CHXD coacervate phase (monomer molar ratio = 2 : 1) was assembled *in situ* within the aqueous lumen of the proteinosomes by addition of CHXD (20 µL, 5 mM in 20 mM Tris-HCl, pH 8) to 20 µL of a centrifuged solution of BSA-NH_2/PNIPAAm proteinosomes containing encapsulated CM-dextran-FITC (10 mM). Similarly, a PDDA/ATP coacervate phase (monomer molar ratio = 1 : 1) was produced inside the proteinosomes by addition of ATP (20 µL, 10 mM in 20 mM Tris-HCl, pH 8) to 20 µL of a centrifuged solution of the proteinosomes comprising encapsulated PDDA (10 mM). In both cases, disassembly of the entrapped coacervate phases was achieved by addition of aqueous NaCl to the proteinosome suspensions (final concentration, 100 mM).

In situ preparation of a PDDA/PAA coacervate (monomer molar ratio = 1 : 1 or 2 : 3 at constant total polymer concentration) within proteinosomes containing PAA, PDDA and NaCl (see above methods) was achieved by dialysis of the crosslinked proteinosomes containing PDDA/PAA at the desired monomer molar ratio against 65, 40 and 20 vol% ethanol/water for 2 h, and then against Milli-Q water for 1 day (see above methods) to remove the NaCl to induce formation of an encapsulated PDDA/PAA coacervate phase and transfer the crosslinked proteinosomes into water.

Enzyme-mediated peroxidation in coacervate-in-proteinosome protocells
Typically, 100 µL of an aqueous dispersion of BSA-NH_2/PNIPAAm proteinosomes (pH 7.5, Tris-HCl (20 mM), NaCl (20 mM)) prepared with encapsulated HRP-FITC (0.8 mg mL^{-1}) and PDDA (10 mM, M_W = 100-200 kDa) was added to a 96-well well plate, followed by addition of ATP (60 µL, 50 mM) to

produce a ATP/PDDA coacervate inside the proteinosomes. An aqueous solution of ABTS (100 µL, 1 mM) was then added and the mixture left for 60 s to ensure that sequestration of the substrate into the coacervate phase had reached equilibrium. A solution of H_2O_2 (40 µL, 1 mM) was then added to initiate the HRP-mediated peroxidation reaction. The increase in absorbance at 410 nm due to formation of the ABTS radical cation (ε410 = 36000 $M^{-1}cm^{-1}$) was recorded as a function of time using a BMG labtech Clariostar plate reader. The enzyme activity in the absence of ATP/PDDA coacervate micro-droplets was carried out following the same procedure except for replacing the ATP solution with Milli-Q water. The kinetic rates were calculated based on the following equation: $[V_0]=\Delta A/\Delta t.\varepsilon.c$, where $\Delta A/\Delta t$ was the change in absorbance at 410 nm over a given time period, ε molar attenuation coefficient of oxidised ABTS (ε410 = 36000 $M^{-1}cm^{-1}$) and c the path length (1 cm).

Enzyme cascade reactions in coacervate-in-proteinosome protocells

Typically, 140 µL of an aqueous suspension (20 mM Tris-HCl, pH 7.5) of proteinosomes comprising a cross-linked membrane of BSA-NH_2/PNIPAAm and HRP-NH_2/PNIPAAm-FITC nanoconjugates (20 and 80 wt%, respectively) and encapsulated GOx-DyLight-405 (0.2 mg mL^{-1}) and PDDA (10 mM, M_W = 100-200 kDa) was added to a 96-well well plate. ATP (25 µL, 100 mM), ABTS (5 µL, 30 mM) and Milli-Q water (20 µL) were then added to produce a thin GOx/ABTS coacervate shell under the HRP-containing membrane. The cascade reaction was initiated by addition of glucose (10 µL, 1.5 mM), and the increase in absorbance at 410 nm due to formation of the ABTS radical cation (ε410 = 36000 $M^{-1}cm^{-1}$) was recorded as a function of time using a BMG labtech Clariostar plate reader. Cascade reactions were also undertaken with the GOx/ABTS partitioned into coacervate droplets dispersed in the proteinosome lumen by adding a mixture containing (25 µL, 100 mM), ABTS (5 µL, 30 mM) and NaCl (20 µL, 300 mM) to the dispersion of HRP-NH_2/PNIPAAm-FITC proteinosomes. The kinetic rates were calculated based on the following equation: $[V_0]=\Delta A/\Delta t.\varepsilon.c$, where $\Delta A/\Delta t$ was the change in absorbance at 410 nm over a given time period, ε molar attenuation coefficient of oxidised ABTS (ε410 = 36000 $M^{-1}cm^{-1}$) and c the path length (1 cm).

General methods

Fluorescent labelling of proteins. Generally, proteins (5 mg) were dissolved in 2.0 mL of pH 8.5 sodium carbonate buffer (100 mM), followed by 50 µL of fluorescein isothiocyanate (FITC) (1.0 mg.mL-1), or ThermoFisher Scientific DyLight-405 (1.0 mg.mL^{-1}) dissolved in DMSO, which were added dropwise. The solution was stirred at room temperature for 5 h, purified by dialyzing against Milli-Q water and freeze dried.

Optical and confocal fluorescence microscopy. Optical microscopy experiments were carried out on a Leica DMI 3000B optical microscope. Fluorescence imaging was performed using a Leica DFC 310FX set up. Confocal fluorescence microscopy measurements were performed using a Leica SP8 AOBS confocal laser scanning microscope attached to a Leica DM I6000 inverted epifluorescence microscope equipped with a resonant scanner and an adaptive focus control to correct focus drift during time-courses with a 65 mW Ar laser (488 nm for FITC excitation, 15% power), 50 mW 405 nm diode laser (DyLight-405 excitation, 10% power). Detection bands were set at 500-560 nm with 20% gain (FITC) and 415-465 nm with 20% gain (DyLight-405). All experiments were carried out in an environmental chamber maintained at 25 °C.

Fluorescence-activated cell sorting (FACS). Freshly prepared dispersions (≈ 1 mL) of CMD-FITC-containing proteinosomes, bulk CMD-FITC/CHXD coacervates (monomer molar ratio = 2 : 1) or coacervate-in-proteinosome protocells (CMD-FITC/CHXD, monomer molar ratio = 2 : 1) were investigated using a FACS Canto II flow cytometer operating at low pressure with a 100 µm sorting nozzle. 2D dot plots of the side-scattered light area (SSC-A) versus forward-scattered light area (FSC-A) were determined for a total of between 10,000-20,000 particles for each population. The samples were analysed immediately after preparation to minimize the effect of coalescence.

Diameter of entrapped CMD-FITC/CHXD coacervate droplets. The diameters of the proteinosome-entrapped CMD-FITC/CHXD (monomer molar ratio = 2 : 1) coacervate droplets were obtained by recording confocal microscopy images approximately in the middle section of individual proteinosomes. The diameters were measured using imageJ image analysis software; typically an average of 100 measurements in groups of approximately 5 individual proteinosomes, with each group representing a specific diameter.

Equilibrium partitioning constant for ABTS. The partition constant (*K*) was determined under equilibrium conditions from $K = C_{COA}/C_S$ where C_{COA} was the concentration of ABTS in a coacervate bulk phase and C_S the concentration of ABTS in the supernatant phase. Typically, 1300 µL of the coacervate micro-droplet solution was prepared by mixing 600 µL of 50 mM PDDA solution with 600 µL of 50 mM ATP solution (both at pH 8) followed by the addition of 100 µL of 10 mM ABTS solution. This solution was left for 10 mins to come to equilibrium and then centrifuged for 15 mins at 800 rpm to separate the bulk and supernatant phases. The concentration of ABTS in each phase was determined by recording the absorbance of ABTS at 340 nm. The ATP/PDDA coacervate phase was disassociated by addition of 0.5 M NaCl prior to UV-Vis analysis to avoid scattering effects associated with the turbid coacervate phase.

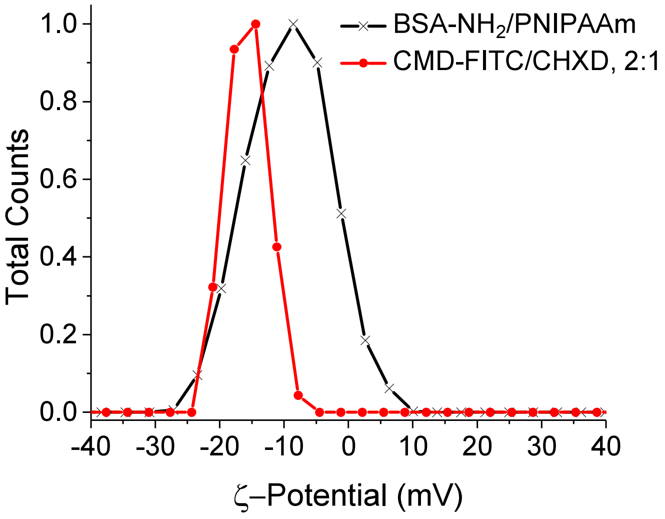

Supplementary Figure S1. ζ-potential measurements for an aqueous solution of $BSA-NH_2$/PNIPAAm nanoconjugates (black line, -9 mV) and CMD-FITC/CHXD coacervate micro-droplets formed in a bulk suspension (red line, -16 mV).

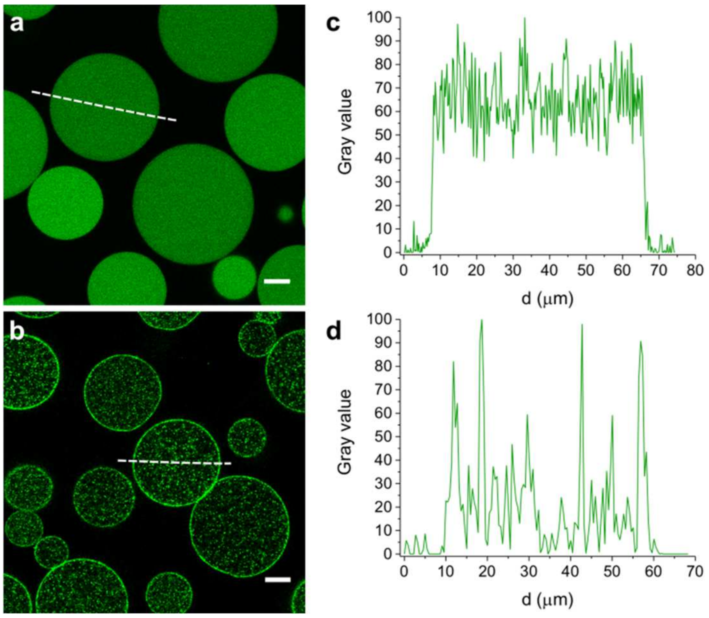

Supplementary Figure S2. (**a,b**) Confocal fluorescence microscopy images of proteinosomes containing encapsulated CMD-FITC showing homogenous green fluorescence before (**a**) or after (**b**) addition of CHXD and subsequent formation of an encapsulated coacervate phase. Scale bars = 20 μm. (**c,d**) Corresponding fluorescence intensity line profiles across single proteinosomes before and after addition of CHXD (dotted lines in **a** and **b**, respectively).

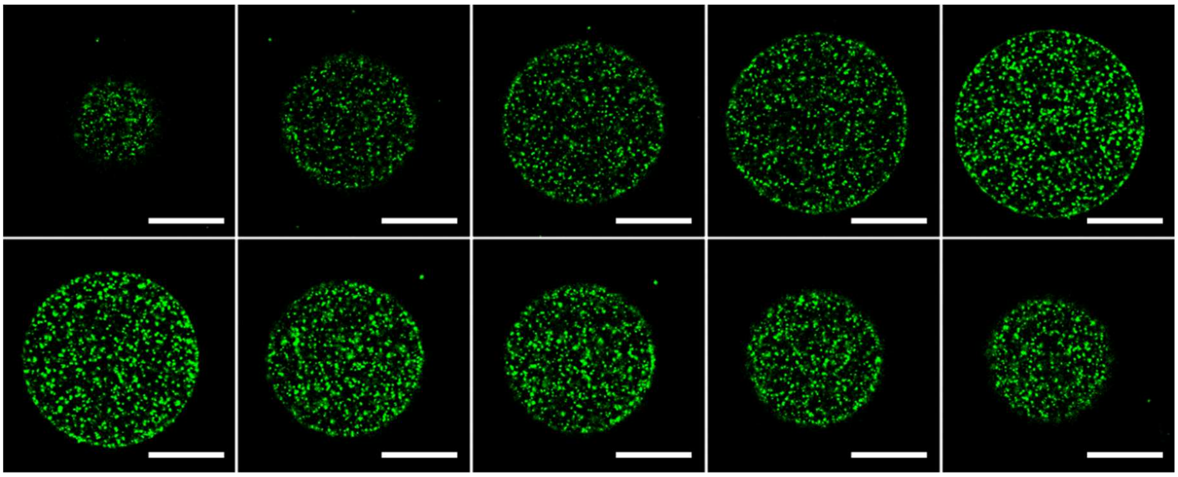

Supplementary Figure S3. (**a-j**) Confocal fluorescence microscopy images of slices across the z-axis of a single BSA-PNIPAAm proteinosome containing an encapsulated CMD-FITC/CHXD coacervate phase showing a dispersed distribution of coacervate micro-droplets within the proteinosome lumen. Optical slices are approximately 5 μm apart, all scale bars = 20 μm.

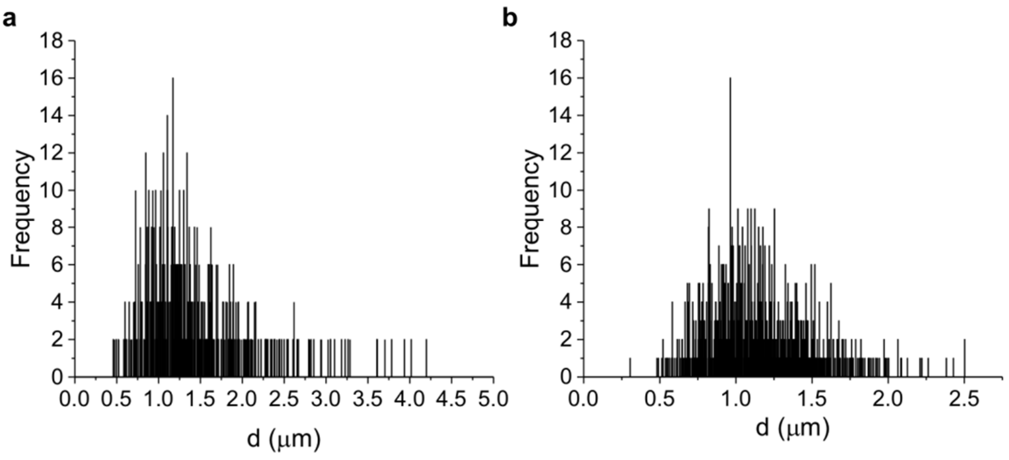

Supplementary Figure S4. (**a,b**) Distribution in the diameter, *d*, of CMD-FITC/CHXD coacervate droplets in the bulk (**a**) and in BSA-NH_2/PNIPAAm proteinosomes (**b**) as measured from confocal fluorescence microscopy images. The diameters of the coacervate droplets in the proteinosomes were measured from optical slices recorded approximately at the centre of the proteinosome microcompartment.

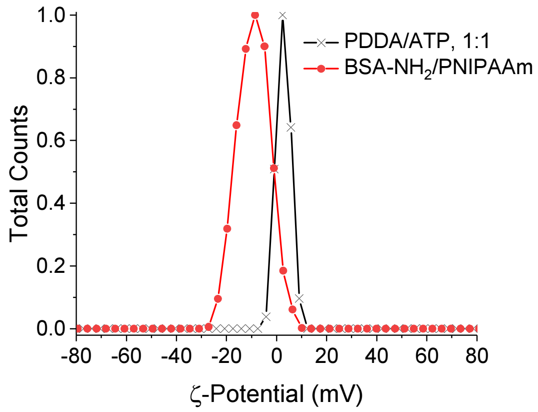

Supplementary Figure S5. ζ-potential measurements for a ATP/PDDA bulk phase coacervate prepared at a 1 : 1 molar ratio (black line, +3 mV) and for an aqueous dispersion of BSA-NH_2/PNIPAAm nanoconjugates (red line, -9 mV).

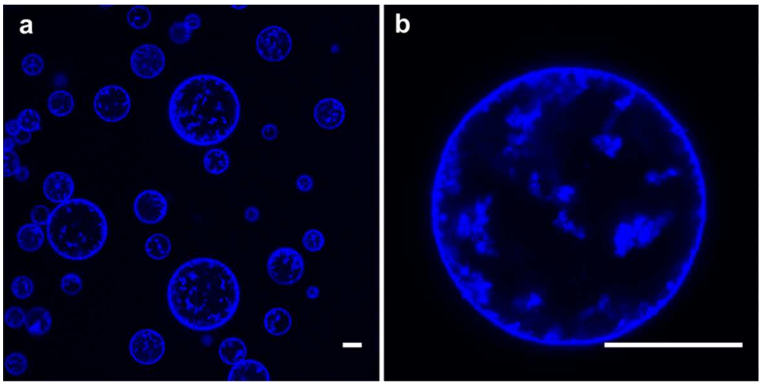

Supplementary Figure S6. (**a,b**) Fluorescence microscopy images of proteinosomes containing a spatially localized ATP/PDDA coacervate phase. Blue fluorescence arises from sequestration of proteinosome-encapsulated GOx-DyLight 405 into the coacervate phase. Scale bars = 20 μm

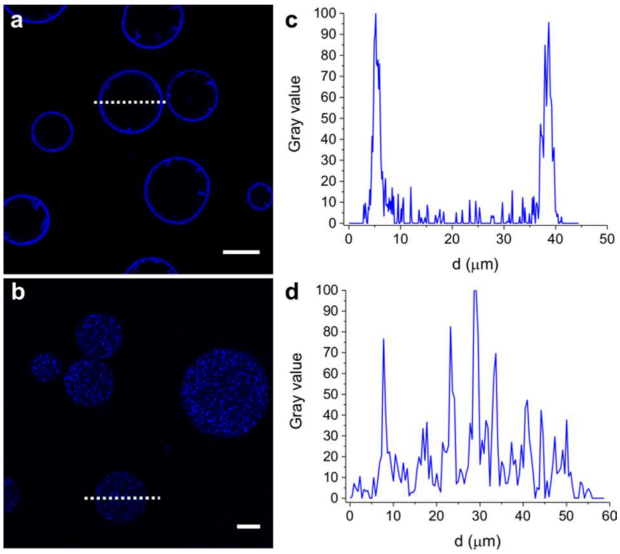

Supplementary Figure S7. (**a,b**) Confocal fluorescence microscopy images of proteinosomes containing a spatially localized ATP/PDDA coacervate phase (**a**), and after addition of NaCl (20 mM) showing transformation to entrapped droplets (**b**). Blue fluorescence arises from sequestration of proteinosome-encapsulated GOx-DyLight 405 into the coacervate phase. Scale bars = 20 μm. (**c,d**) Corresponding fluorescence intensity line profiles across single proteinosomes shown in **a** and **b** (dotted lines) confirming the different spatial arrangements before and after addition of NaCl.

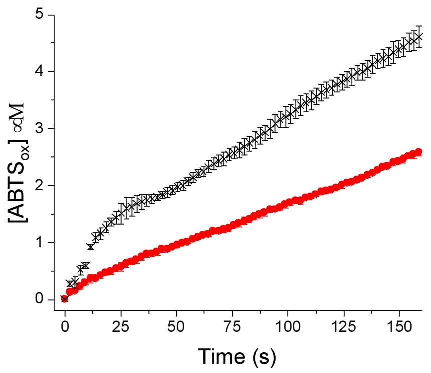

Supplementary Figure S8. Time profile showing increase in [$ABTS_{ox}$] within proteinosomes containing HRP-loaded ATP/PDDA coacervate micro-droplets (black) or in the absence of the coacervate phase (red) over a period of 160 s.

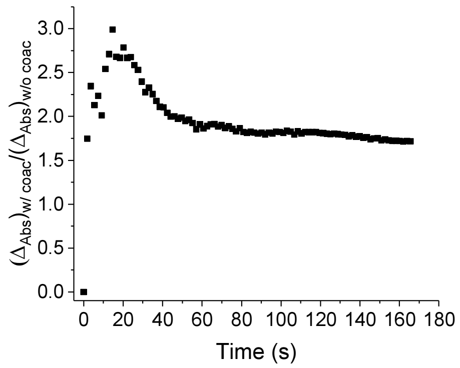

Supplementary Figure S9. Time profile of the relative differences in changes in absorbance for the proteinosome-located HRP-mediated one-election oxidation of ABTS in the presence $(\Delta A_{abs})_{w/coac}$ or absence $(\Delta A_{abs})_{w/o\ coac}$ of encapsulated ATP/PDDA coacervate microdroplets.

Supplementary Notes

Phase transformation-mediated assembly of coacervate-in-proteinosome protocells

Host-guest nested protocells were also produced by co-encapsulation of high ionic strength (0.5 M NaCl) solutions of poly(acrylic acid) (PAA) and PDDA in the cross-linked proteinosomes followed by removal of the salt to induce coacervate phase transformation by lowering of the charge screening. By controlling the macromolecular molar ratios and hence the surface charge on the incipient coacervate primary droplets (**Supplementary Note: Figure S01**), a few or multiple coacervate droplets could be produced inside the proteinosomes depending on their stability with regard to coalescence **Supplementary Note: Figure S02a**). A dense population of discrete droplets was generated in the presence of excess PAA (PAA : PDDA monomer molar ratios = 3 : 2 (**Supplementary**

Note: Figure S02b,c), whilst a single large coacervate droplet or a few droplets were produced within the proteinosomes under charge neutral conditions (**Supplementary Note: Figure S02d,e**). In the former, the entrapped droplets were stable to coalescence over a period of at least 30 d (**Supplementary Note: Supplementary Figure S03**). In contrast, the single coacervate micro-droplets were essentially stationary and resided predominately in the centre of the sedimented proteinosomes, presumably due to their higher density compared with the surrounding aqueous solution. In both cases, co-encapsulation of the polyelectrolytes with DyLight 405-labelled glucose oxidase (GOx) indicated that enzymes in the aqueous interior of the proteinosomes could be efficiently partitioned into the PAA/PDDA coacervate droplets (**Supplementary Note: Figure S02c,e**). Assembly of the droplets within the proteinosomes was reversible such that addition of aqueous HCl (pH 4.5) or NaCl (0.5 M) resulted in fast dissolution of the coacervate phase (**Supplementary Note: Supplementary Figure S04**).

Supplementary Note: Methods: Disassembly of entrapped PAA/PDDA coacervates. PAA/PDDA coacervate micro-droplets were prepared at a monomer molar ratio of 3 : 2 within BSA-NH_2/PNIPAAm proteinosomes by dilution-mediated phase transformation. 40 μL of the aqueous dispersion of nested protocells was mounted on an optical microscopy slide and 2 μL of 5 M HCl added to decrease the pH to ca. 4.5 to protonate the acrylic acid groups of PAA. Alternatively, 10 μL of a 5 M solution of NaCl was added (final concentration = 0.5 M). Both procedures resulted in disassembly of the entrapped coacervate phase.

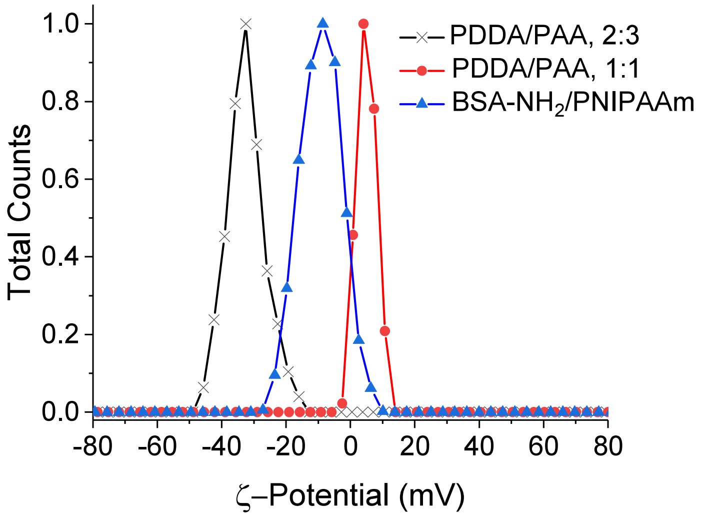

Supplementary Note Figure S01. ζ-potential measurements for a PAA/PDDA bulk phase coacervate prepared at a PAA : PDDA molar ratio of 3 : 2 (black line, -33 mV) and at 1 : 1 (red line, +4 mV). ζ-potential measurements for an aqueous solution of BSA-NH_2/PNIPAAm nanoconjugates are also shown (blue line, -9 mV).

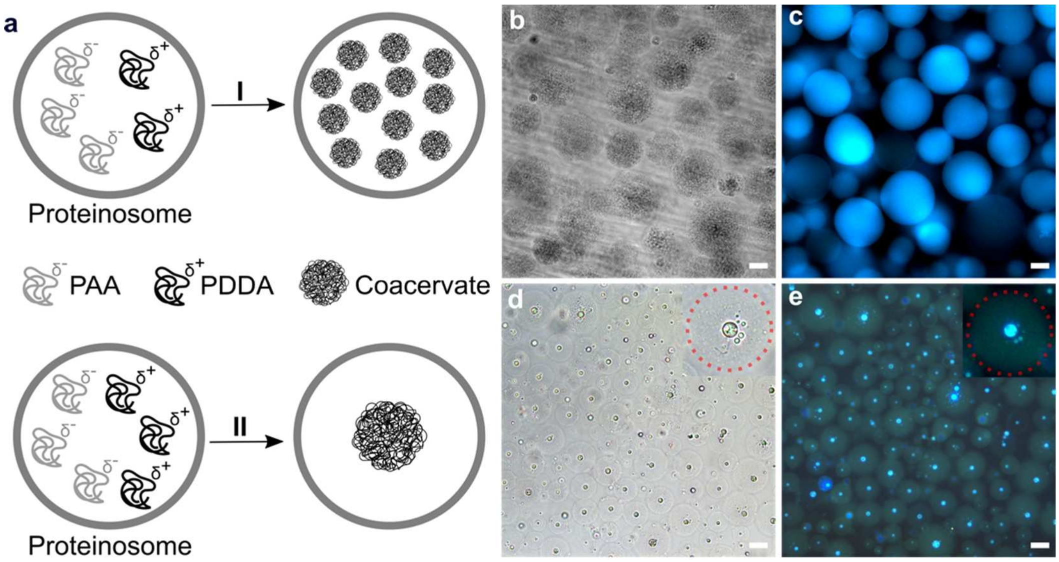

Supplementary Note Figure S02. Phase transformation-mediated assembly of coacervate-in-proteinosome protocells. (**a**) Scheme illustrating two phase transformation-mediated assembly pathways (**I** and **II**) used to generate proteinosomes with multiple- or single-trapped coacervate micro-droplets produced at PAA : PDDA monomer molar ratios of 3 : 2 (**I**) or 1 : 1 (**II**), respectively. (**b-e**) Optical (**b,d**) and fluorescence (**c,e**) microscopy images of coacervate-in-proteinosome protocells prepared via pathway **I** (**b,c**) or **II** (**d,e**), respectively. A dense population of non-coalesced droplets is formed via pathway **I**, whilst coalescence dominates in pathway **II** to give only a few large droplets in each proteinosome. The coacervate phase is stained with encapsulated GOx-DyLight 405 (blue fluorescence). Insets in **d** and **e** show a single proteinosome with a single centrally positioned coacervate droplet and a few minor satellite droplets. Scale bars = 20 μm.

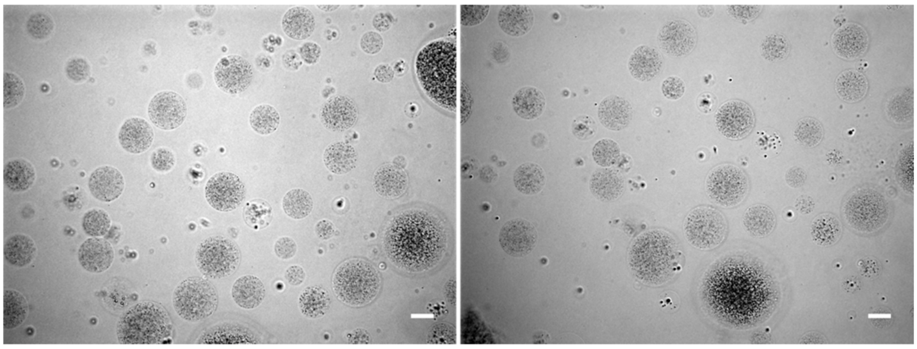

Supplementary Note Figure S03. Optical microscopy images of proteinosomes containing a dense population of highly negatively charged PDDA/PAA coacervate droplets prepared at a PAA : PDDA molar ratio of 3 : 2) showing minimal coalescence after a period of 30 d. Scale bars = 20 μm.

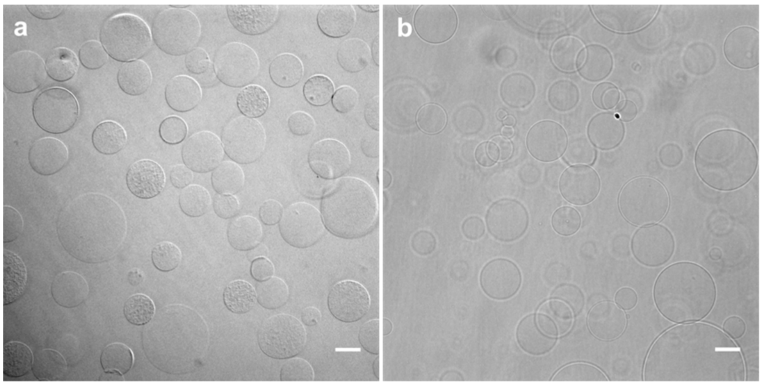

Supplementary Note Figure S04. (**a,b**) Optical microscopy images of PAA/PDDA (molar ratio = 3 : 2) coacervate-containing proteinosomes after acidification (addition of HCl) (**a**), or after an increase in ionic strength (addition of NaCl; final concentration = 500 mM) (**b**). In both cases, the entrapped coacervate micro-droplets are disassembled within 10 s to produce optically transparent proteinosomes. Scale bars = 20 μm.
